# Enhanced Dielectric Performance in PVDF-Based Composites by Introducing a Transition Interface

**DOI:** 10.3390/polym17020137

**Published:** 2025-01-08

**Authors:** Congcong Zhu, Kun Li, Xiaoxu Liu, Yanpeng Li, Jinghua Yin, Lu Hong, Qibing Qin

**Affiliations:** 1 School of Computer Engineering, Weifang University, Weifang 261061, China; zhucc6@163.com (C.Z.); qinbing@wfu.edu.cn (Q.Q.); 2 School of Material Science and Engineering, Shaanxi University of Science and Technology, Xi’an 710021, China; 3 School of Electrical Engineering, Yancheng Institute of Technology, Yancheng 224007, China; lypharbin@163.com; 4 Key Laboratory of Engineering Dielectrics and Its Application, Ministry of Education, Harbin University of Science and Technology, Harbin 150080, China

**Keywords:** PVDF-based composites, titanium oxide nanosheets, aluminum oxide transition interface, microstructure, dielectric performance

## Abstract

Polymeric dielectrics have garnered significant interest worldwide due to their excellent comprehensive performance. However, developing polymeric dielectric films with high permittivity (*ε*_r_) and breakdown strength (*E*_b_) and low dielectric loss (tan*δ*) presents a huge challenge. In this study, amorphous aluminum oxide (Al_2_O_3_, AO) transition interfaces with nanoscale thickness were constructed between titanium oxide (TiO_2_, TO) nanosheets and polyvinylidene fluoride (PVDF) to manufacture composites (PVDF/TO@AO). TO@AO nanosheets showed favorable dispersion in the polymer-based composites. Improved permittivity, suppressed dielectric loss, and enhanced breakdown strength were achieved by introducing AO coating with intermediate permittivity onto TO nanosheets to build a transition interface. The transition interface efficiently depressed the mobility of the charge carrier and electric conduction of the PVDF/TO@AO composites. As a result, the PVDF-based composite with 1 wt% TO@AO showed superior comprehensive performance, including high *ε*_r_ of ~12.7, low tan*δ* of ~0.017, and exceptional *E*_b_ of ~357 kV/mm. This strategy supplies a novel paradigm for the application of fabricating dielectric films with excellent comprehensive performance.

## 1. Introduction

Polymer dielectric materials have the characteristics of good flexibility, strong chemical inertness, good insulation, and low raw material cost. They have wide potential in the areas of long-distance direct current transmission, dual power-source vehicles, solid oxide fuel cells, and pulsed power systems [1,2,3,4,5]. However, most polymers have low permittivity, and the development of polymeric dielectric films with high permittivity, low dielectric loss, and high breakdown strength still presents great challenges in industry and academia [6,7].

Introducing high permittivity nanofillers into polymer-based composites is the most efficient manner to improve the permittivity of polymer-based composite dielectrics [8,9,10,11]. The advantages of transition metal oxides, such as high permittivity, good electrical insulation, strong interface interaction, and good thermal stability, make them ideal fillers for improving the dielectric insulation properties of polymer composite films. These properties complement each other and can significantly improve the application potential of polymer composites in electrical and electronic fields, especially in the development of high-permittivity materials with excellent insulation properties [12,13,14,15,16]. Wang et al. used zinc oxide nanoparticles (ZnO) as filler to prepare PVDF-based composite films. The permittivity of 3.45 wt% PVDF/ZnO composite films was as high as 21 at 10^3^ Hz [17]. However, large dielectric difference between nanofillers and organic matrix results in deformation of the electric field, and it is difficult for the polymer-based composite to maintain excellent dielectric insulation performance. The construction of an insulating shell with appropriate permittivity can optimize the electric field distribution, inhibit the mobility of free electrons, limit the electric field distortion, and relieve the mismatching of dielectric properties in the interface region [18,19].

Furthermore, by introducing a medium-permittivity shell between high-permittivity nanofillers and a low-permittivity matrix, an insulation structure with gradient distribution of dielectric parameters (permittivity/conductivity) gradient distribution can be constructed. The distribution of the electric field is regulated by the distribution of dielectric parameters, alleviating the excessive local field strength and homogenizing the electric field’s distribution, thereby significantly improving the composite’s dielectric properties [20,21]. Recent studies have shown that the permittivity and breakdown strength can be improved by introducing a TiO_2_ shell that regulates the permittivity, which can be attributed to the construction of a structure with gradient distribution. A composite with 5 vol% BT@TiO_2_ exhibited superior comprehensive performance, including a high *ε*_r_ of ~12.7, a low tan*δ* of ~0.037, and an exceptional *E*_b_ of ~277 kV/mm [22]. Theoretical and experimental results show that compared with 0D or 1D nanofillers, 2D nanosheets with high dielectric content have a high aspect ratio and a large dipole moment, which tends to enhance the permittivity and breakdown strength of polymer-based composite dielectrics with low doping content [23,24]. However, because of its high surface energy, the free volume at the interface increases, and the interface compatibility between the filler and the matrix is poor, which deteriorates the dielectric properties of the composite. Furthermore, the large differences in permittivity and conductivity between the 2D nanofiller and the polymer matrix usually lead to a significant deterioration of the breakdown strength of the composite dielectrics. Interface engineering by constructing shell structures on the surface of inorganic nanosheets is an effective strategy to solve the above problems [25,26,27].

In the current study, AO transition interfaces were constructed between TO nanosheets and PVDF to fabricate PVDF/TO@AO composites, leading to a gradient distribution of permittivity between the nanosheets and polymer matrix. The TO@AO nanosheets were well dispersed in the PVDF matrix. The effect of microstructures on the performance of the PVDF/TO@AO composites was explored. The results showed that the composites possessed high permittivity, low dielectric loss, and excellent breakdown strength compared with pristine PVDF and PVDF/TO composites. Therefore, this work presents a novel method that involves introducing transition interfaces to fabricate composites with good comprehensive dielectric performances.

## 2. Materials and Methods

### 2.1. Materials

PVDF powder was purchased from Sigma-Aldrich Co., Ltd. (Shanghai, China). K_2_CO_3_, Li_2_CO_3_, TiO_2_, and tetrabutylammonium hydroxide (TBAOH, AR) were obtained from Aladdin (Shanghai, China). N, N-dimethylformamide (DMF, AR) was obtained from Tianjin Fuyu Chemical Co., Ltd. (Tianjin, China), CH_5_NO_2_ (analytical reagent), CH_2_O_2_ (analytical reagent), and Al_2_(SO_4_)_3_·18H_2_O (analytical reagent) were obtained from Sinopharm Chemical Reagent Co., Ltd. (Shanghai, China).

### 2.2. Preparation of TO@AO Nanosheets

**Figure 1:**
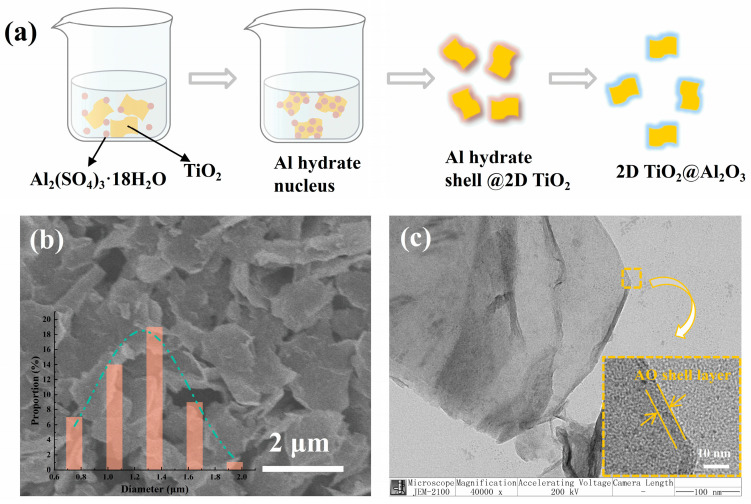
(**a**) Fabrication of TO@AO nanosheets; (**b**) SEM results for TO@AO (inset the lateral size distribution); (**c**) TEM image of AO@TO nanosheet with inset showing the high-resolution TEM image.

TiO_2_ nanosheets were synthesized via the top-down exfoliation method, as described in the Supplementary Materials [28]. For this purpose, 6.3 g of ammonium formate was dissolved in 500 mL deionized water, and formic acid was added to adjust the pH to 4.6. Then, 1 g of TiO_2_ and 3.2 mM aluminum sulfate were added. The mixture was heated at 70 °C with stirring for 2 h and then centrifuged. Finally, the samples were heated at 300 °C for 2 h to obtain TO@AO nanosheets. In this study, it was assumed that AO was mainly formed on the TO surface during the fabrication process. A schematic illustration of the fabrication process of TO@AO nanosheets can be seen in Figure 1a.

### 2.3. Fabrication of PVDF/TO@AO Composite Films

Firstly, TO or TO@AO nanosheets with different mass fractions were ultrasounded in 7 mL DMF solvent then mixed with 1 g PVDF powder and stirred for 12 h. The mixture was left for 12 h until it was free of air bubbles then poured on a clean glass and coated with a scraping plate. The sample was dried in an oven at 80 °C for 10 h, hot pressed at 180 °C and 15 MPa under pressure by a plate vulcanizer, and then kept for 30 min. The thickness of the obtained composite film was about 20 ± 5 μm.

### 2.4. Characterization

The structures of the TO@AO nanosheets and composites were measured by SEM (Hitachi SU8020, Tokyo, Japan) and TEM (JEM-2100, Tokyo, Japan). The phase structures of the TO@AO nanosheets and composites were studied by XRD using a Bruker D8 Advance device (Buker, Hong Kong, China) with a scan range (2*θ*) of 5–80°. The molecular valence bonds of the TO@AO nanosheets and composite films were investigated by FTIR using JASCO 6100 (Jasco Inc., Easton, MD, USA). Small-angle X-ray scattering (SAXS) was tested on a 1W2A beamline at the Synchrotron Radiation Facility in Beijing. The thermal transition data of composite films were tested by differential scanning calorimetry (DSC). A DSC3 MultiSTARe system produced by METTler Toledo (Greifensee, Switzerland) was used to perform DSC testing on the composite films. The ambient gas was set as nitrogen, the test temperature for the heating section was set at 40 °C–180 °C, and the heating rate was set as 10 °C/min. The permittivity and related parameters of composites were studied using a wideband impedance analyzer (GmbH Novocontrol Alpha-A, Novocontrol Technologies GmbH & Co. KG, Montabaur, Germany). The breakdown strength and leakage current density at 10 Hz were tested by YDZ-560 (Chengdu Kelaisi Liquid Nitrogen Container Co., Ltd., Chengdu, China).

### 2.5. Finite Element Method (FEM) Simulations

Finite element method (FEM) simulations were employed to explore the electric field distribution of the PVDF/TO and PVDF/TO@AO composites, where the nanofillers were introduced randomly into the polymer matrix. In this simulation, the electrostatic field of AC/DC module was selected as the physical field, and the two-dimensional cross-sectional plane was used to build the model. A voltage was applied between the top and bottom of the simulation system, decreasing from 1 kV to 0 kV.

## 3. Results

### 3.1. Characterization of TO@AO Nanosheets

The morphology of TO@AO nanosheets was characterized by SEM, as shown in Figure 1b. The 2D nanosheets showed typical wrinkled and silk-like morphology. The inset of Figure 1b demonstrates the statistical size distribution of the nanosheets, based on counting about 50 samples. The average lateral size of the TO@AO as obtained was around 0.6–2.8 µm. A TEM image of the TO@AO core-shell structured nanosheets and high-resolution TEM image of interfaces between TiO_2_ and Al_2_O_3_ are displayed in Figure 1c and the inset of Figure 1c, respectively. The TO@AO nanosheets possessed a clear core–shell nanostructure, and the AO shell layer was homogenous and dense.

**Figure 2:**
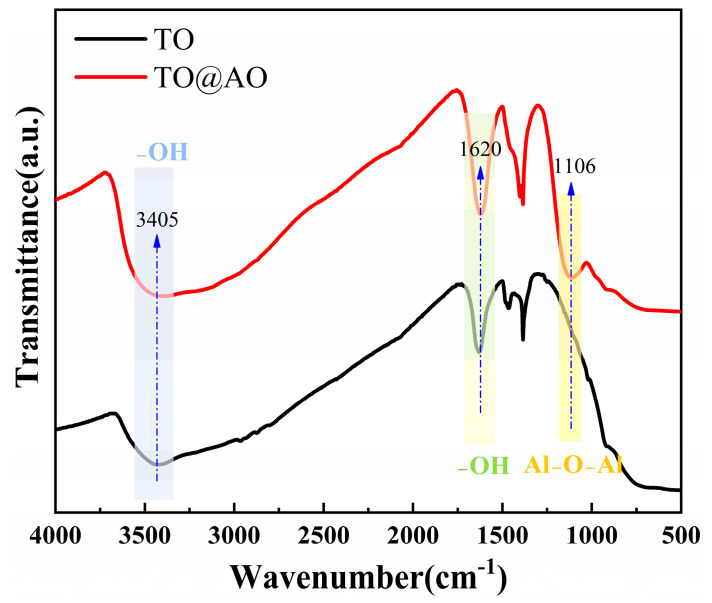
FTIR spectra of TO and TO@AO nanosheets.

Figure 2 shows the FTIR spectra of the TO and TO@AO nanosheets. The peaks at 3405 cm^−1^ and 1620 cm^−1^ were attributed to the –OH stretching and bending vibrations, respectively, and were present on both TO and TO@AO nanosheets. Additionally, the peak at 1106 cm^−1^, corresponding to the Al-O stretching vibration, confirmed the presence of an AO shell on the TO nanosheets, consistent with the TEM results [29].

### 3.2. Characterization of PVDF/TO@AO Composites

**Figure 3:**
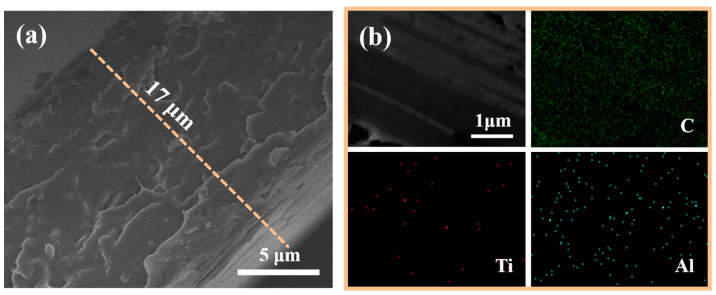
(**a**) Cross-sectional SEM image of the PVDF/TO@AO composites with 1 wt%; (**b**) cross-sectional TEM image and corresponding elemental mapping of PVDF/TO@AO composite with 1 wt% TO@AO.

The cross-sectional morphology of the 1 wt% composite is shown in Figure 3a. The TO@AO nanosheets showed homogeneous dispersion without large agglomerates or the formation of defects. In the image, the interface between PVDF and TO@AO is unclear and hard to distinguish, suggesting strong compatibility. Figure 3b shows the cross-sectional TEM image and corresponding elemental mapping of the 1 wt% composite. The uniform distributions of Ti and Al elements suggest a homogeneous distribution of 2D nanofillers and a dense overall structure. Some TO nanosheet surfaces may not have been completely covered with AO or the coating may be thin, resulting in some Ti elements still being detected.

**Figure 4:**
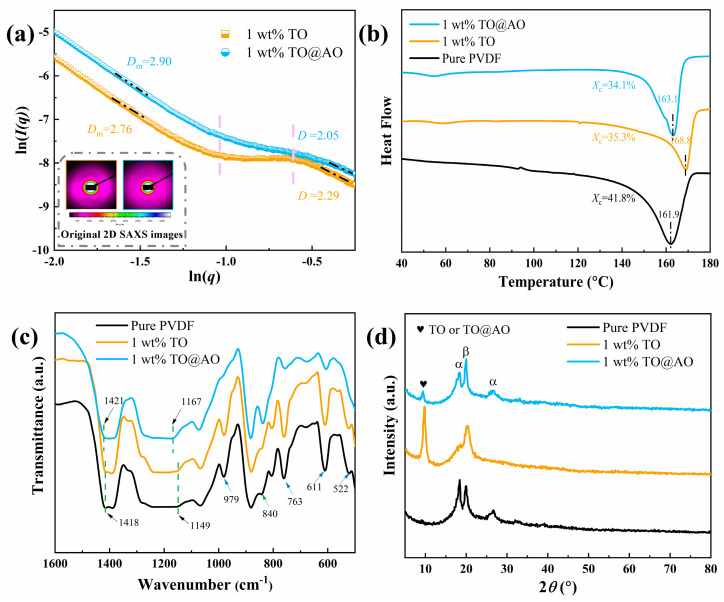
Schemes follow the same formatting. (**a**) The original 2D SAXS images and the ln(q) − ln(I(q)) plots, (**b**) DSC endothermic curves, (**c**) FTIR, and (**d**) XRD results for all three kinds of composites.

SAXS was applied to further explore the microstructure of the PVDF/TO and PVDF/TO@AO composite films; the original 2D SAXS images are presented in the inset of Figure 4a. One important parameter derived from the SAXS analysis was the fractal dimension (*D*), critical for characterizing the overall structural complexity and morphology of polymer-based composites. The fractal dimension provides information on the self-similarity and the level of intricacy in the material’s microstructure, making it a valuable tool for evaluating the degree of ordering or disordering in composite systems [30]. *D* can be calculated from ln[*I*(*q*)] − ln(*q*) plots following *I*(*q*) = *q*^−D^. The ln[*I*(*q*)] − ln(*q*) plots of different composites with 1 wt% nanofillers are depicted in Figure 4a. It is well known that the dispersed phase exhibits a one-dimensional fibrous morphology when *D* = 1, while *D* = 2 represents a two-dimensional plate morphology, and *D* = 4 represents a spherical morphology [31]. The *D* of both composites was approximately 2 near the relatively high *q* region, and the value of *D* indicated the presence of the TO and TO@AO nanosheets. The mass fractal (*D*_m_) dimension of the composites at the low *q* region reflected the compactness of the composites. The *D*_m_ of the PVDF/TO composite was 2.76, whereas the *D*_m_ of the PVDF/TO@AO reached 2.90. The increased value of *D*_m_ indicated that the PVDF/TO@AO composite had a relatively compact dense microstructure and smaller free volume compared with the PVDF/TO composite.

The crystallization of the nanocomposites was investigated by DSC analysis. As shown in Figure 4b and Figure S1, the incorporation of TO or TO@AO increased the melting temperature (*T*_m_) of the PVDF matrix. The crystallinity (Xc) of the pristine PVDF was 41.8%, which was much higher than that of the composites containing 1 wt% TO (35.3%) and the 1 wt% TO@AO nanosheets (34.1%). This change was mainly due to the strong interaction between the nanosheets and the PVDF chain. Specifically, the introduction of nanosheets may somehow have impeded the free movement of the PVDF molecular chains, thereby limiting crystal growth. In particular, the 1 wt% PVDF/TO@AO composite had a lower Xc value compared with the pristine PVDF and 1 wt% PVDF/TO composites, which can be attributed to the stronger interaction between the AO layer and the PVDF chain. The aluminum hydroxyl group on the AO layer further inhibited the arrangement and crystallization of the PVDF chain by forming hydrogen bonds with the fluorine atoms of the PVDF; so, the crystallinity of the composite with the AO transition interface was further reduced [32].

Figure 4c and Figure S2 display the FTIR spectra of all samples, with the absorption peaks at 1418 and 1149 cm^−1^ corresponding to the bending vibration of CH_2_ and stretching vibration of the C-F bond, respectively. The incorporation of the TO nanosheets did not change the position of CH_2_ and C-F absorption peaks, indicating the weak interfacial interaction between the PVDF chains and TO nanosheets. However, apparent changes in the absorption peaks of the PVDF/TO@AO composites were observed. The absorption peaks of the CH_2_ and C-F bonds in the composites with 1 wt% TO@AO nanosheets shifted toward higher wavenumbers, which indicated that the AO layer formed a strong interaction with the PVDF molecules [33]. In addition, the absorption peaks located at 522, 611, 763, and 979 cm^−1^ matched the α-phase PVDF. The peaks positioned at 840 cm^−1^ were associated with *β*-phase PVDF. Similar absorption peaks were observed, indicating that the *α* phase and *β* phase coexisted in all three kinds of composites. The XRD spectra of all the samples are depicted in Figure 4d and Figure S3. The unique (020) plane belonging to the nanosheets is clearly identified. At 2*θ* = 18.5°, 26.7°, and 20.2°, the diffraction peaks were attributed to the (020) plane, the (021) plane of the *α* phase, and the (110) plane of the *β* phase, respectively [34]. The α phase and β phase were observed in the pure polymers and the PVDF/TO and PVDF/TO@AO composites. The addition of 2D nanofillers with different structures was found to have a negligible effect on the crystalline structure of the PVDF.

### 3.3. Dielectric Properties of PVDF/TO@AO Composites

**Figure 5:**
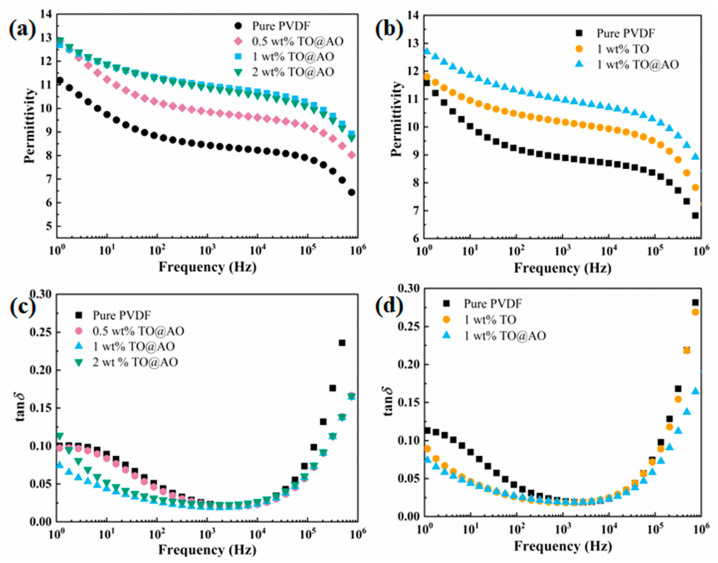
(**a**,**b**) Dielectric permittivity and (**c**,**d**) dielectric loss tangent of all three kinds of composites.

The permittivity (*ε*_r_) of composites filled with TO@AO is shown in Figure 5a. With the increasing mass fraction of TO@AO, the *ε*_r_ of the PVDF/TO@AO composites increased and then decreased. For comparison, the *ε*_r_ of the composite filled with TO was also measured, as displayed in Figure 5b. The composite with 1 wt% TO@AO exhibited the highest *ε*_r_ of 11.3 at 10^2^ Hz, which was 9.1% higher than that of the PVDF/TO composite (10.5). Compared with the PVDF/TO composite, the PVDF/TO@AO composite showed higher permittivity, which may be ascribed to the enhanced interface polarization brought on by the introduction of transition interfaces [35]. The reason for the decrease in the permittivity of the 2 wt% PVDF/TO@AO composite may be that at high doping content, the overlapping of nanosheets led to a reduction in the interface area, thereby weakening the interface polarization. Figure 5b displays the tan*δ* of the PVDF/TO@AO composites. The tan*δ* of the PVDF/TO@AO composites showed an obvious opposite trend to the *ε*_r_, with the mass fraction of TO@AO increasing. The tan*δ* of the PVDF/TO@AO composites decreased firstly and then increased; the minimum of 0.07 is achieved when 1 wt% TO@AO was incorporated at 1 Hz. Remarkably, the PVDF/TO@AO composite showed significantly decreased values, as shown in Figure 5d. Figures S4 and S5 display the imaginary part of the electric modulus (*M*″) of the pristine PVDF and composites. Compared with the 1 wt% PVDF/TO composite, the trough of the spectrum shifted towards a higher frequency for the 1 wt% PVDF/TO@AO composite, reflecting the amplified impact of interfacial polarization [36]. Based on the *M*″ of the composites shown in Figure S5, it can be inferred that the AO transition interface contributed to the additional interfacial polarization, thereby reducing conduction loss. The PVDF/TO@AO composites exhibited both high *ε*_r_ and low tan*δ* values.

**Figure 6:**
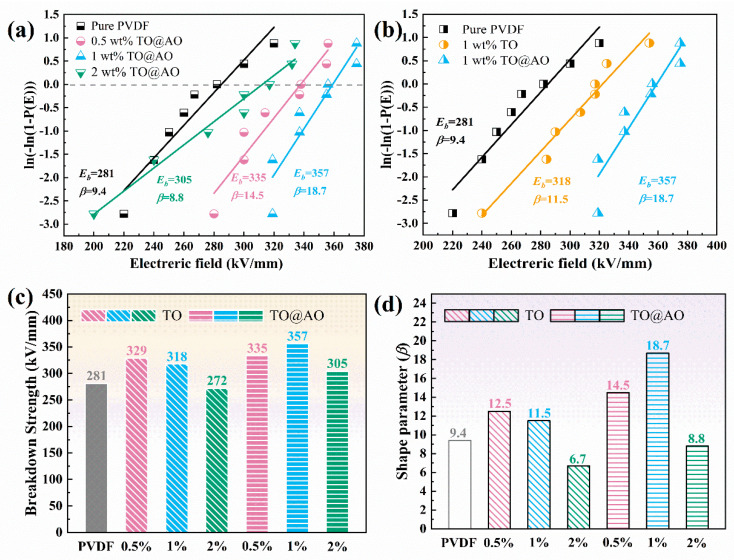
(**a**,**b**) Two−parameter Weibull distribution, (**c**) *E*_b_, and (**d**) *β* of all three kinds of composites.

Two−parameter Weibull distribution was used to explore the breakdown strength of composites with various mass fractions of nanofillers [37]. The cumulative probability of electric failure *P* can be described as follows:

*P* = 1 − exp[−(*E*_b_/*E*)*^β^*], (1)

where *E*_b_ is the tested breakdown strength, *E* denotes the breakdown strength when the cumulative failure probability reaches 63.2%, and *β* means a shape parameter, which reflects the scattering of *E*_b_. Typically, a higher *β* value corresponds to a more concentrated distribution of breakdown strength, resulting in greater reliability of the composite film. Figure 6a,b depict the two-parameter Weibull distribution of *E*_b_ of all the samples. Figure 6c,d show the *E*_b_ and *β* of all the samples. As the TO@AO mass fraction increased, the *E*_b_ of the PVDF/TO@AO composites initially rose and then dropped. Especially, the 1 wt% PVDF/TO@AO composite showed a notable improvement in *E*_b_ when compared with the other samples, as depicted in Figure 6c. Specifically, the 1 wt% PVDF/TO@AO composite possessed an excellent *E*_b_ of 357 kV/mm, which was 27.05% and 12.26% higher than the values for the pristine PVDF (281 kV/mm) and 1 wt% PVDF/TO composite (318 kV/mm). Furthermore, as illustrated in Figure 6d, the PVDF/TO@AO composites possessed higher *β* than the other comparison samples, indicating the superior reliability and quality of the composites [38]. The increased breakdown strength of the PVDF/TO@AO composites can be attributed to the ability of AO transition interface to inhibit carrier migration, which significantly helped reduce the conduction loss in the composites, thereby preventing breakdown failure.

**Figure 7:**
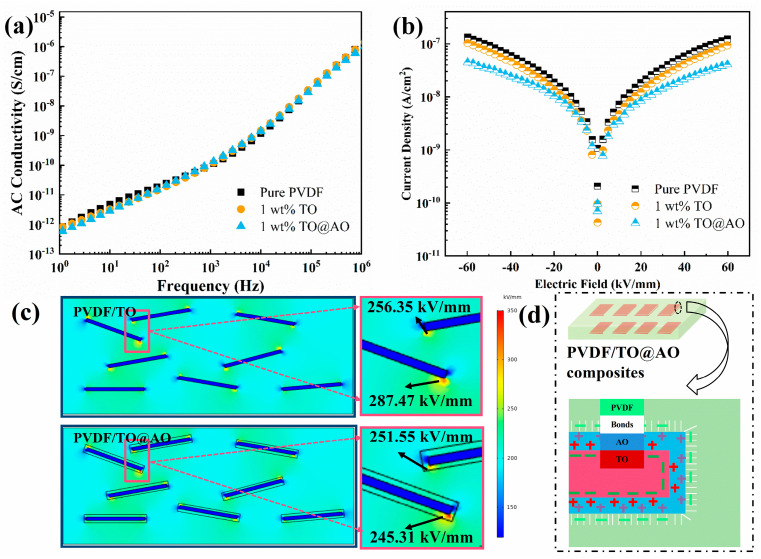
(**a**) The AC conductivity and (**b**) current density of all three kinds of composites. (**c**) Finite element simulation of local electric field for PVDF/TO and PVDF/TO@AO composites. (**d**) Diagram of charge accumulation at TO–AO and AO–PVDF interface.

To explore the reasons for the improvement in the *E*_b_ of the composites, the AC conductivity and leakage current were tested as shown in Figure 7a,b. The 1 wt% PVDF/TO@AO composite exhibited low AC conductivity and leakage current, indicating that the introduction of the AO transition interface between the TO filler and PVDF matrix inhibited carrier transport and conduction. To further investigate the influence of the AO transition interface on the local electric field of the composite, the electric field of the composites with TO nanosheets and TO@AO nanosheets as nanofillers was investigated via the finite element method. The huge dielectric difference between the high-permittivity nanofiller and the low-permittivity polymer can cause local electric field distortion, resulting in a decrease in breakdown strength. In the simulation, the permittivity of the TO was set to 40, AO to 10, and PVDF to 8. Figure 7c shows the finite element simulation results for the electric field distribution of the PVDF/TO and PVDF/TO@AO composites. The colors in the figure reflect the strength of the local electric field. The local electric field’s distortion at the interface of the PVDF/TO@AO composites was weaker than that of the PVDF/TO composites. The AO transition layer with moderate permittivity was able to optimize the electric field distribution in the composite, inhibit the free electron migration, limit the electric field distortion, and alleviate the dielectric mismatch in the interface region. Figure 7d presents a diagram of charge accumulation at the TO-AO and AO-PVDF interface. These interface charges form a Gouy–Chapman–Stern layer on the TO surface, which has high electrical conductivity, allowing charges to move freely within it [25]. If the nanofiller layers overlap, the free charge may migrate over a shorter distance, triggering a larger leakage current and reducing the *E*_b_. Nevertheless, the strong interaction between the AO transition interface and matrix promotes the uniform dispersion of nanosheets and inhibits the establishment of current channels, thus preventing the generation of leakage current [39,40].

## 4. Conclusions

In summary, PVDF-based composites were developed using 2D core-shell structure TO@AO nanosheets as nanofillers and PVDF in the role of matrix. An AO shell layer with moderate permittivity was coated onto the TO nanosheets’s surface to construct a transition interface area, leading to a gradient distribution of permittivity between the nanosheets and the matrix. Furthermore, the *ε*_r_ and *E*_b_ of the composites was significantly improved after the introduction of the transition interface. On the one hand, the construction of the transition interface introduced more interfacial regions inside the composites, resulting in enhanced interfacial polarization and thus, increased permittivity. On the other hand, the construction of a transition interface with permittivity gradient distribution was able to inhibit the mobility of free electrons, alleviate the dielectric mismatch in the interface region, and cause low dielectric loss and an increase in breakdown strength. As a result, the composite filled with 1 wt% TO@AO had a superior permittivity of 12.7, a low dielectric loss of 0.017, and an excellent breakdown strength of 357 kV/mm. This work has demonstrated that introducing transition interfaces to the manufacture of composites can contribute to excellent comprehensive dielectric performance.

## Data Availability

Data are contained within the article and Supplementary Materials.

## Supplementary Materials

The following supporting information can be downloaded at: https://www.mdpi.com/article/10.3390/polym17020137/s1, Figure S1: The DSC endothermic curves of pure PVDF and PVDF/TO@AO composites; Figure S2: The FTIR spectra of pure PVDF and PVDF/TO@AO composites; Figure S3: The XRD patterns of pure PVDF and PVDF/TO@AO composites; Figure S4: The electric modulus imaginary part (M″) of pure PVDF and PVDF/TO@AO composites; Figure S5: The electric modulus imaginary part (M″) of pure PVDF, PVDF/TO and PVDF/TO@AO composites; Figure S6: The AC conductivity of pure PVDF and PVDF/TO@AO composites.
