# Supplementary material for: Enhanced Dielectric Performance in PVDF-Based Composites by Introducing a Transition Interface
Source: Polymers (Basel). 2025 Jan 8;17(2):137. doi: 10.3390/polym17020137 (PMC11768189; doi:10.3390/polym17020137)
Supplement: Supplementary file 1 [file polymers-17-00137-s001.zip › polymers-3330333-supplementary.pdf]

Supporting Information

Enhanced dielectric performance in PVDF-based composites by introducing transition interface

Congcong Zhu[1], Kun Li*[1], Xiaoxu Liu[2], Yanpeng Li[3], Jinghua Yin[4] and Lu Hong[1]
[1] School of Computer Engineering, Weifang University, Weifang, 261061, P.R. China.
[2] School of Material Science and Engineering, Shaanxi University of Science and Technology, Xi'an, 710021, P.R. China.
[3] School of Electrical Engineering, Yancheng Institute of Technology, Yancheng, 224007, P.R. China.
[4] Key Laboratory of Engineering Dielectrics and Its Application, Ministry of Education, Harbin University of Science and Technology, Harbin 150080, P. R. China.
Correspondence to：*E-mail: 20220082@wfu.edu.cn

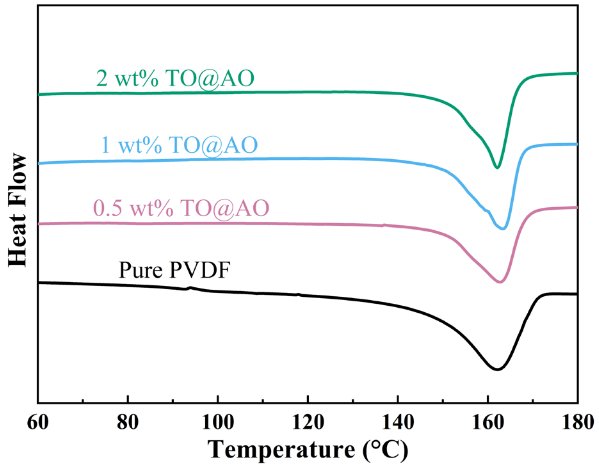

Figure S1. The DSC endothermic curves of pure PVDF and PVDF/TO@AO composites.

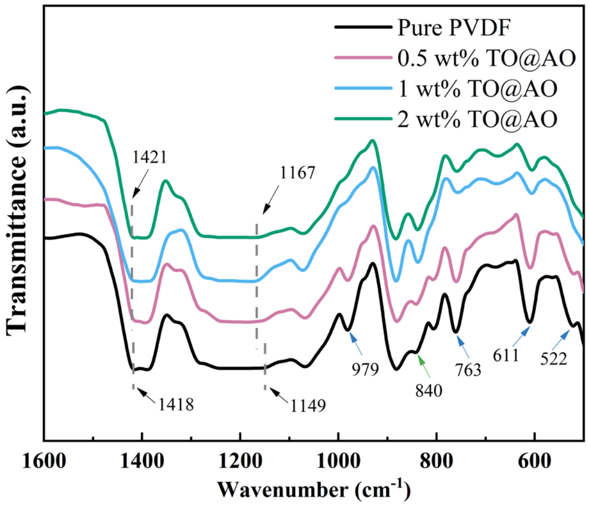

Figure S2. The FTIR spectra of pure PVDF and PVDF/TO@AO composites.

.

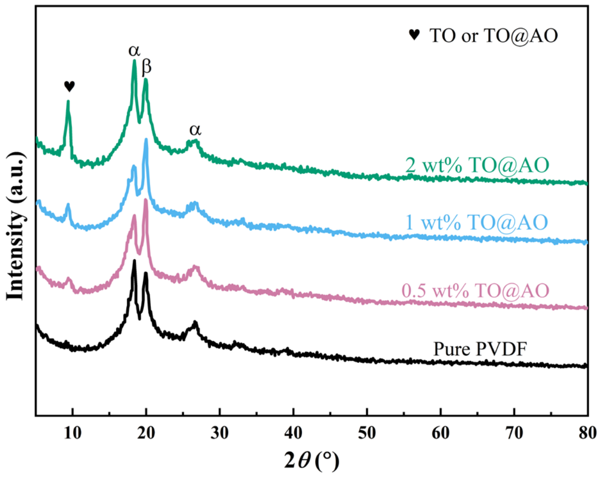

Figure S3. The XRD patterns of pure PVDF and PVDF/TO@AO composites.

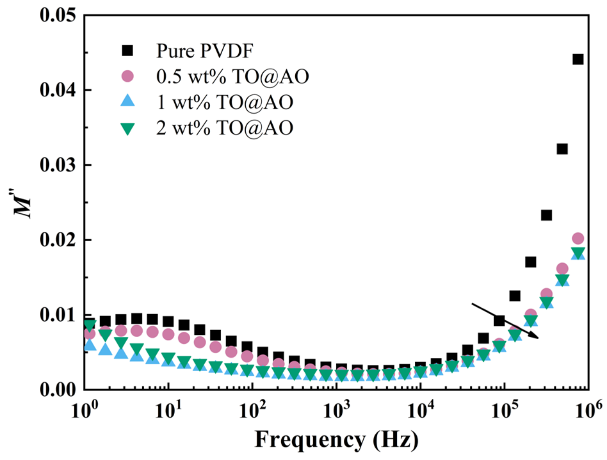

Figure S4. The electric modulus imaginary part (M'') of pure PVDF and PVDF/TO@AO composites.

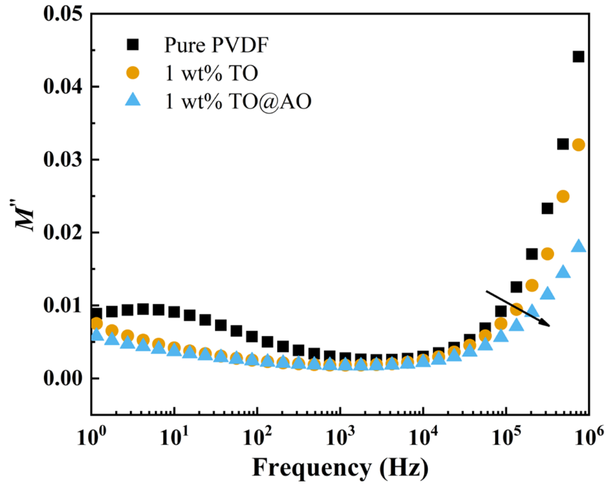

Figure S5. The electric modulus imaginary part (M'') of pure PVDF, PVDF/TO and PVDF/TO@AO composites.

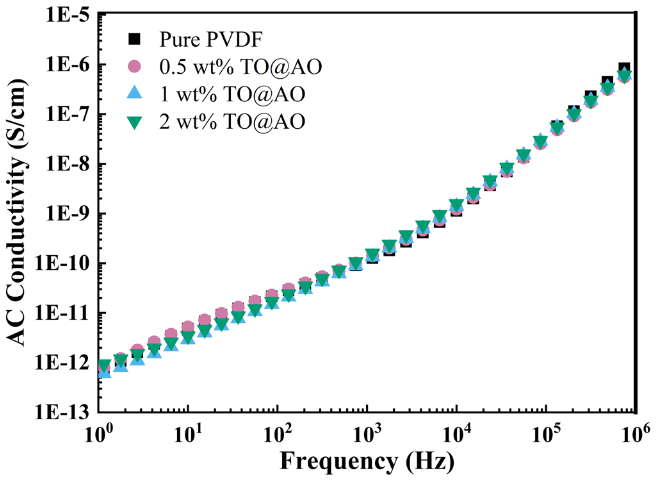

Figure S6. The AC conductivity of pure PVDF and PVDF/TO@AO composites.
